# Fiber Optic Sensor of Axial Strain and Dynamic Transverse Force Based on Intensity Demodulation

**DOI:** 10.3390/s25247441

**Published:** 2025-12-07

**Authors:** Cezary Kaczmarek, Malgorzata Detka

**Affiliations:** 1 Department of Electronics and Information Technology, Lublin University of Technology, Nadbystrzycka 38A, 20-618 Lublin, Poland; 2 Department of Computer Science, Electronics and Electrical Engineering, Kielce University of Technology, Aleja TysiącleciaPaństwaPolskiego 7, 25-314 Kielce, Poland; m.detka@tu.kielce.pl

**Keywords:** fiber sensor, intensity demodulation, dynamic force, strain

## Abstract

This paper presents a fiber-optic sensor with intensity demodulation for simultaneous measurement of dynamic transverse force and axial strain. The sensor uses a Sagnac loop filter with a polarization-maintaining photonic crystal fiber (PM-PCF) that is subjected to a dynamic transverse force. The Sagnac loop filter is illuminated by the reflected beam froma uniform fiber-optic Bragg grating (FBG), which is subjected to an axial strain. This way, intensity demodulation is performed in the sensor, enabling it to measure two quantities simultaneously: the dynamic force and the strain. Experimental results show that the sensor achieves a sensitivity to the dynamic transverse force of 38.1 mV/N and a sensitivity to the axial strain of 0.527 mV/με, while the nonlinearity errorsare 4.9% for the dynamic force and 0.9% for the strain. The sensor exhibits low temperature sensitivity due to partial self-compensation of the temperature coefficients of the Sagnac loop filter with the polarization-maintaining photonic crystal fiber and the fiber Bragg grating.

## 1. Introduction

Force and strain are quantities whose measurement is of great importance in many fields of technology, medicine, and scientific research. Recently, various types of fiber-optic sensors for these physical quantities have been increasingly used in these measurements due to their known advantages. The predominant sensitive components of these sensors are fiber-optic Bragg gratings (FBGs), polarization-maintaining fibers (PMFs), conventional single-mode and multi-mode fibers, and fiber-optic elements such as couplers.

An important issue in fiber-optic sensor technology is the simultaneous measurement of two or more quantities using one sensor. Sensors that enable this type of measurement arecalled dual-parameter or multi-parameter sensors. For this purpose, different wavelengths and/or different propagation modes are usually used, for which the sensitivities of the transducers to the measured quantities are different. When determining the sensitivities ofthe measured quantities todifferent wavelengths or different propagation modes, the processing equation of a multi-parameter sensor, written in matrix form, makes it possible to reconstruct the measured quantities by calculating the inverse of the sensitivity matrix, provided that the determinant of the sensitivity matrix has a value other than zero.

The pairs of quantities most frequently measured simultaneously are strain and temperature [1,2,3], refractive index and temperature [4,5,6], liquid level and temperature [7,8,9], and force and temperature [10,11,12]. The three quantities most frequently measured simultaneously are torsion, strain, and temperature [13,14,15]. In recent publications on simultaneous strain and temperature measurement using FBG sensors, the authors have applied new machine learning models, including artificial neural networks and regression models. They achieved improved accuracy and higher measurement resolution compared to the standard inverse sensitivity matrix method [16,17,18]. The above-mentioned multi-parameter measurements are based on wavelength demodulation and are most often measurements of quasi-static quantities. There is often a need to simultaneously measure a time-varying and a quasi-static quantity.

This paper presents a dual-parameter sensor for a simultaneous measurement of strain and time-varying transverse force using intensity demodulation. The sensor is based on a Sagnac loop filter (SLF) with a polarization-maintaining photonic crystal fiber (PM-PCF), which is illuminated by the reflected beam froma uniform fiber Bragg grating (FBG). The varying transverse force acting on the PM-PCF of the SLF induces synchronous shifts in its spectral transmission, which, in turn, cause changes in the intensity of the filter’s output beam. The tensile strain of the FBG shifts the spectrum of its reflected beam, resulting in a change in the constant component of the SLF output beam intensity.

The novelties of the proposed setup compared to previous works include the possibility fora simultaneous measurement of a dynamic force and a quasi-static strain, adding a second function for the FBG as a strain transducer alongside its primary function as an arrow band light source for the SLF, dual parameter separation via DC/AC decomposition, and the absence of cross-sensitivities between the measured variables, as long as the setup operates in the linear range of the SLF.

## 2. Experimental Setup and Principle of Operation

**Figure 1:**
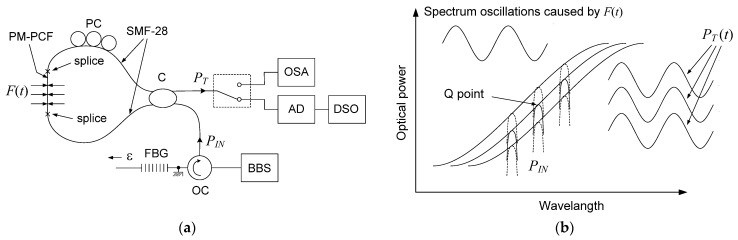
Schematic diagram of the (**a**) experimental setup, and (**b**) mechanism for dynamic two-parameter sensing.

**Figure 2:**
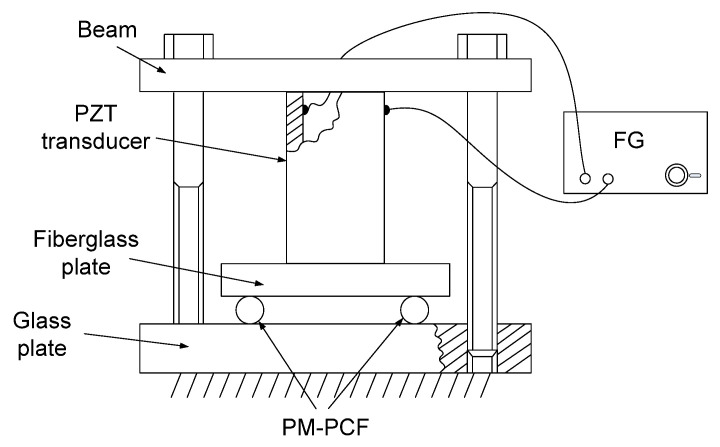
Device for generating a transverse compressive force acting on the optical fiber.

The experimental setup of the proposed two-parameter sensor of strain and dynamic transverse force is shown in Figure 1a. A beam from an amplified spontaneous emission (ASE) broadband source (BBS), model BBS 1550-TS from AFC Technologies Inc. of Hull, Quebec, Canada, illuminates a uniform FBG with a Bragg wavelength of 1.5502 μm using an optical circulator (OC). The reflected beam from the grating is fed to the SLF via the circulator and its coupler (C). A 0.354 m long segment of a polarization-maintaining photonic crystal fiber (PM-PCF), model PM-1550-01 from Thorlabs of Newton, New Jersey, USA, is installed in the loop of the SLF as the transverse force-sensitive element. The transmission beam of the SLF is converted to a voltage by an amplified photodetector (AD), model PDA400 from Thorlabs of Newton, New Jersey, USA,, and recorded with a digital storage oscilloscope (DSO), model GDS 1054B from GW INSTEK of New Taipei City, Taian. The polarization controller (PC) in the filter loop is used to improve the filter’s extinction ratio and set its operating point. The optical spectrum analyzer (OSA), together with the optical switch (OS), enables the observation of the setting of the SLF’s operating point and the determination of its sensitivity to strain and transverse force. The principle of the two-parameter processing with intensity detection in the sensor is schematically shown in Figure 1b. A sinusoidally varying transverse force acting on the PM-PCF of the SLF causes synchronous shifts in its spectral transmission, which modulate the power of the grating’s reflected beam at the transmission output of the filter. The tensile strain of the FBG causes a shift in the spectrum of its reflected beam and, therefore, a change in the operating point of the SLF, which results in a change in the constant component of the power of this beam at the transmission output of the filter. To generate a variable transverse force acting on the PM-PCF, a piezoelectric transducer (PZT), model Pz27 from Meggitt A/S, was used, controlled by the voltage output from a function generator (FG), model JC5603P from NDN. The PZT was installed in compression mode, as shown in Figure 2.

In the intensity demodulation method, the fundamental requirement is the stability of the light source output. Changes in the light source power directly affect the output signal power, leading to measurement errors. To eliminate this effect, the well-known method of using the power ratio (*P_T_* − *P_R_*)/(*P_T_* + *P_R_*) as the sensor output signal can be employed. This requires feeding the reflected beam *P_R_* out of the Sagnac loop, which requires either using a second 3-port circulator in the current system or replacing the current 3-port circulator with a 4-port circulator and supplementing the current system with a second signal-processing path, including a second amplified photodetector.

The phase difference between the polarization modes propagating in a polarization-maintaining fiber is given by

(1) δ=2πLB/λ,

where *B*, *L*, and *λ* denote the PMF’s phase birefringence, the PMF’s length, and the wavelength, respectively.

The spectral transmission of the SLF is related to the group birefringence *G* of the PMF, as indicated by Equation (2) obtained after differentiating Equation (1) and performing simple transformations.

(2) $$d\delta /d\lambda =-2\pi LG/\lambda^{2}.$$

If one neglects the losses in the 3 dB coupler and the attenuation of the PMF and the single-mode fiber (SMF) in the Sagnac loop, the spectral transmission of the SLF is approximately a periodic function of wavelength, namely [19,20]

(3) $$T=\left[1-\cos\left(2\pi LB/\lambda \right)\right]/2.$$

The period of the transmission spectrum Λ, i.e., the wavelength spacing between successive transmission peaks or dips, is given by Λ = *λ*^2^/(*GL*). The change in the phase difference Δ*δ* induced by the action of the transverse force on the PM-PCF, assuming that the elongation of the optical fiber can be neglected, can be written approximately as follows:

(4) $$\Delta \delta =\frac{2\pi L}{\lambda }\Delta B,$$

where Δ*B* =Δ*n_x_* − Δ*n_y_* is the change in the phase modal birefringence of the PM-PCF induced by the photo-elastic effect.

A transverse force applied to an optical fiber induces compressive stresses along the axis of the force and tensile stresses along the perpendicular axis. When the stress region is longer than the diameter of the fiber, the stresses in the axial direction will be canceled [21].

The application of the transverse force to a PM fiber can increase or decrease its initial birefringence depending on the orientation of the applied force, which causes rotation of the principal axes of the fiber [22]. The result of the action of the transverse force on the PM fiber is the rotation of its polarization axes in accordance with the principal strain directions [23]. Therefore, the orientation of the polarization axes is a function of the magnitude of the force and the direction in which it is acting. If the transverse force acts on the PM fiber parallel to its fast polarization axis, its phase birefringence increases, shifting the SLF spectrum towards higher wavelengths, reaching a maximum spectrum shift for a given force. When the force is deviated from the previous direction, both clockwise and counterclockwise, in a plane perpendicular to the longitudinal axis of the optical fiber while maintaining the initial force value, the increase in the phase birefringence will decrease until it reaches zero, at an angle of deviation of the force direction from the direction of the fast polarization axis of ±45. As the deviation angle increases, the fiber’s phase birefringence decreases, causing a spectral shift towards lower wavelengths. The maximum decrease in the phase birefringence of the fiber will occur when the directions of the force and the slow axis of the fiber coincide, causing the maximum shift in the SLF spectrum. If the PM fiber is compressed along its slow polarization axis, its birefringence decreases, shifting the SLF spectrum toward lower wavelengths.

Taking into account the relationship between the transmission spectrum shift Δ*λ_d_* and the change in the phase difference Δ*δ*, i.e., Δ*λ_d_* = (Λ/2*π*)·Δ*δ*, and using Equation (4), the spectrum shift Δ*λ_d_* caused by the action of the transverse force on the PM-PCF can be written as

(5) $$\Delta \lambda_{d}=\left(\Lambda L/\lambda \right)\Delta B.$$

where *λ_d_* is the wavelength of a selected dip.

The measured sensitivity of the SLF to the compressive force *F* that acts along the fast polarization axis of its PM-PCF is Δ*λ_d_*/*F* = 0.1 nm/N. The axial strain *ε* of the FBG induces a shift in its Bragg wavelength Δ*λ_B_*, as described by the known relationship.

(6) $$\Delta \lambda_{B}=\lambda_{B}(1-p_{e})\varepsilon ,$$

where *p_e_* is the effective strain-optic constant, and *λ_B_* is the Bragg wavelength. The strain sensitivity of the employed FBG is Δ*λ_B_*/*ε* = 1.2 nm/mε.

The transmission spectrum of the SLF oscillates in synchrony with changes in the transverse force acting on its PMF. The transmission as a function of time *T*(*t*) for the Bragg wavelength of the reflected FBG beam, in the case of a varying transverse force *F*(*t*) acting on the PMF of the SLF, can be written as follows:

(7) $$T\left(t\right)=T_{0}\left(\varepsilon \right)+\frac{dT}{dF}F\left(t\right),$$

where *T*_0_(*ε*) = *T*_0_ + (*dT*/*dε*)·*ε* is the transmission of the SLF at the Bragg wavelength of the reflected FBG beam, dependent on the grating’s tensile strain *ε*, *dT*/*dF* is the intensity sensitivity, defined as the change in transmission induced by a force of unit amplitude, and *dT*/*dε* is the intensity sensitivity, defined by the change in transmission induced by a strain of the FBG of unit amplitude.

The constant component of the output voltage of the photodetection system contains information about the tensile strain of the FBG, while the variable component carries information about the variable transverse force acting on the PMF of the SLF. Intensity sensitivities *dT*/*dF* and *dT*/*dε* can be written as

(8) $$\frac{dT}{dF}=\frac{dT}{d\lambda }\cdot \frac{d\lambda_{d}}{dF},$$

(9) $$\frac{dT}{d\varepsilon }=\frac{dT}{d\lambda }\cdot \frac{d\lambda_{B}}{d\varepsilon }.$$

The first term inEquations (8) and (9), *dT*/*dλ*, is the slope of the transmission spectrum of the SLF for the central wavelength of the reflected beam of the FBG. Its value can be determined from transmission spectrum measurements or analytically. The second terms inthese equations, *dλ_d_*/*dF* and *dλ_B_*/*dε*, are wavelength sensitivities, defined as the shifts in the transmission spectrum induced by a unit force and a unit strain, respectively. The values of these sensitivities can be determined from measurements. The slope of the transmission spectrum of SLF can be determined based on Equation (3) and is expressed by the equation.

(10) $$\frac{dT}{d\lambda }==-\frac{\pi LG}{\lambda^{2}}\sin\left(\frac{2\pi LB}{\lambda }\right).$$

Taking into account the formula for the period Λ in Equation (10), the slope of the transmission spectrum at the quadrature points is obtained.

(11) $${\left(\frac{dT}{d\lambda }\right)}_{Q}=\pm \frac{\pi }{\Lambda }.$$

The plus sign in Equation (11) refers to the rising edges and the minus sign to the falling edges of the transmission spectrum. This value can be easily changed by changing the PMF length in the loop of the SLF: the longer this length, the higher the value of (*dT*/*dλ*)*_Q_* and vice versa. This way, the *dT*/*dF* and *dT*/*dε* sensitivities can be changed. For the employed PM-PCF, model PM-1550-01, at *λ* = 1.55 μm, the measured value of Λ = (7.92 ± 0.05) nm, and thus (*dT*/*dλ*)*_Q_* = 0.4/nm. Therefore, (*dT*/*dF*)*_Q_* = 0.04/N, and (*dT*/*dε*)*_Q_* = 0.42/mε.

## 3. Measurement Results and Discussion

**Figure 3:**
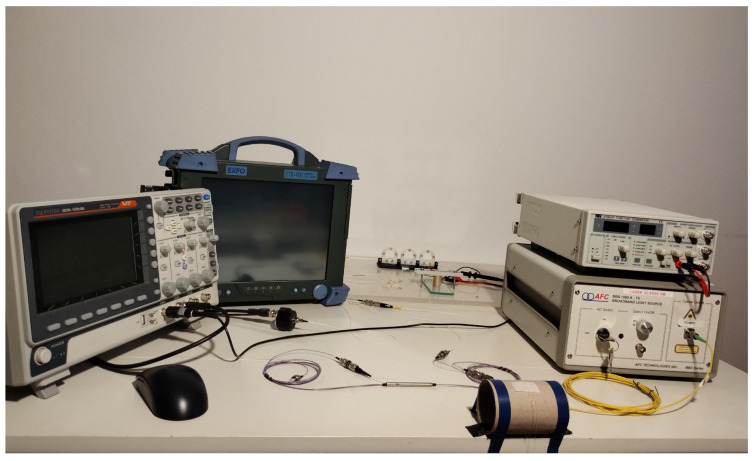
Photograph of the measurement setup.

**Figure 4:**
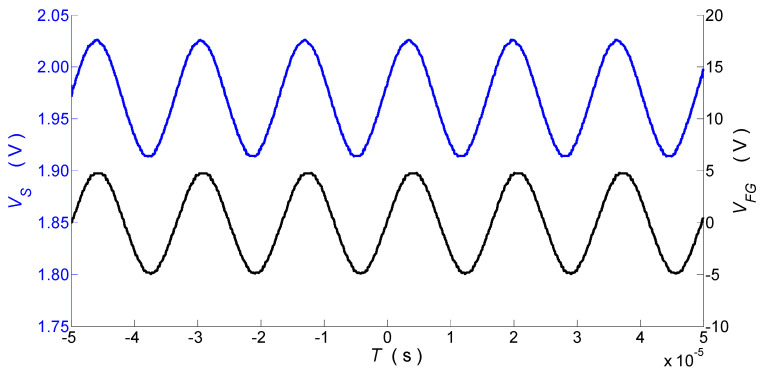
Output voltage of the sensor—left y axis (blue line). Output voltage of the function generator controlling the PZT—right y axis (black line).

The simultaneous measurements of the variable transverse force acting on the PM-PCF and the tensile strain of the uniform FBG were performed in the setup shown in Figure 1a. A photograph of the measurement setup is shown in Figure 3. The piezoelectric transducer installed in the device, as shown in Figure 2, operates in compression mode at its longitudinal resonance frequency, enabling the use of low control voltages. Figure 4 shows the time waveforms of the sensor’s output voltage and the FG’s output voltage controlling the piezoelectric transducer PZT, recorded by the DSO. The variable component of the sensor output voltage corresponds to a sinusoidally varying force with an amplitude of 1.5 N and has the same frequency as the PZT control signal. The observed phase shift between these waveforms is due to detuning from the PZT resonance.

**Figure 5:**
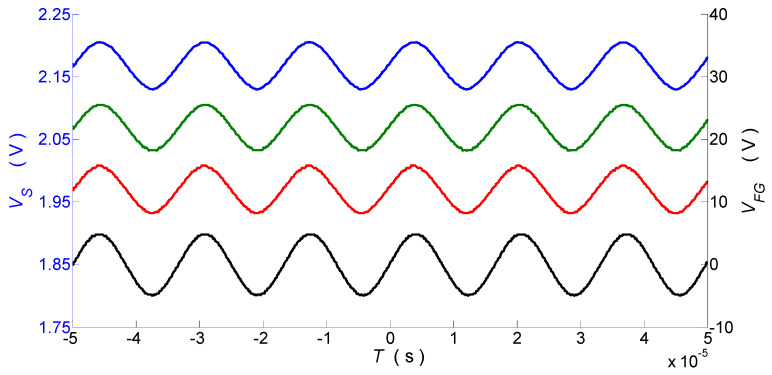
Output voltage of the sensor (left y axis), caused by a sinusoidal PM-PCF compressing force with an amplitude of 1 N for three FBG strain values: 0 με (red line), 224 με (green line), and 448 με (blue line). Output voltage of the function generator controlling the PZT: right y axis, black line.

The time waveform of the sensor output voltage corresponding to the action of a sinusoidally varying force compressing the PM-PCF with a frequency of 60.225 kHz and an amplitude of 1.0 N, for three values of tensile strain of the FBG: 0 με, 224 με, and 448 με, is shown in Figure 5. The operating point of the SLF was chosen on the rising edge of its spectral transmission, as shown in Figure 1b. The initial operating point was set at a distance of 270 pm from the quadrature point Q towards the shorter wavelengths, so that with the FBG deformation of 448 με, the operating point would move along the transmission slope to the other side of the Q point at a distance of 270 pm towards the longer wavelengths. In this way, the linear part of the spectral transmission is optimally used, and minimal nonlinearity of the sensor transfer function is achieved.

**Figure 6:**
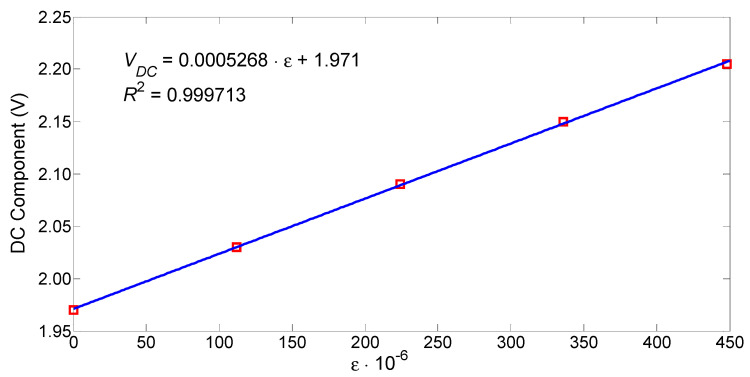
Constant component of the output voltage as a function of the tensile strain of the FBG.

**Figure 7:**
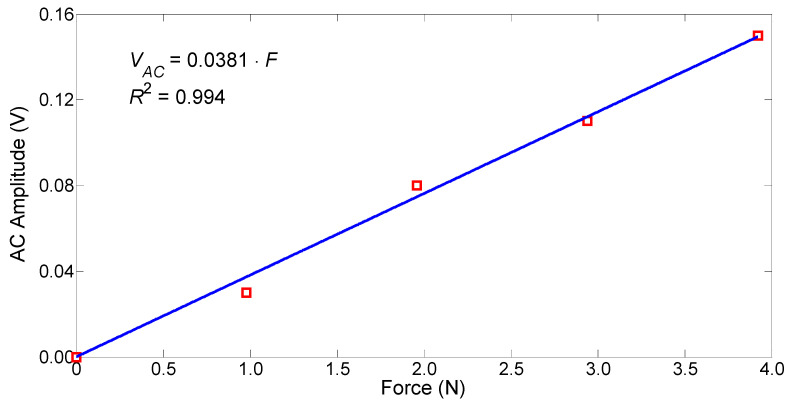
Amplitude of the variable component of the output voltage as a function of the transverse force compressing the PM-PCF.

The constant component of the sensor output voltage as a function of the FBG strain is shown in Figure 6, and the amplitude of the variable component of this voltage as a function of the variable transverse force compressing the PM-PCF is shown in Figure 7. These functions were obtained from a static calibration of the sensor. The strain of the FBG was induced by applying a load to the freely hanging portion of the fiber containing the FBG, while the portion of the fiber without the FBG was clamped to a table. Weights of 10 g and 20 g were used for loading. Loading the FBG with a 10 g weight corresponds to its tensile strain of 112 με. The FBG was strained over the range 0–448 με with a step of 112 με, while the constant component of the sensor output voltage was measured. The transverse load on the PM-PCF was applied using 100 g and 200 g weights placedcentrally on the beam above the PZT (Figure 2). The optical fiber was loaded in the range of 0–3.96 N with a step of 0.98 N, while the increase in the constant component of the sensor output voltage was determined.

A linear curve fitting applied to the sensor’s transfer function shows that its sensitivity to strain and transverse force is 0.527 mV/με and 38.1 mV/N. The values of the correlation coefficient indicate a good fit of the straight line to the measurement results. The determined nonlinearity error of these functions is 0.9% and 4.9%, respectively. The dynamic ranges for the force and strain measurements are 40 dB and 46 dB, respectively.

Knowing the value of the correlation coefficient *R*, which was determined when fitting the straight line to *n* measurement points, the standard uncertainty of the measurement of the strain sensitivity *s_ε_* and the force, and the standard uncertainty of the measurement of the sensitivity *s_F_* of the sensor werecalculated, based on the formula [24,25]:

(12) $$u\left(s_{\varepsilon ,F}\right)=\left|s_{\varepsilon ,F}\right|\sqrt{\frac{\left(1/R^{2}\right)-1}{n-2}}.$$

The following values of these uncertainties were obtained: *u*(*s_ε_*) = 51·10^−7^ V/με and *u*(*s_F_*) = 17·10^−4^ V/N.

The variable force applied to the PM-PCF does not affect the FBG’s strain;therefore, it has no effect on the DC component of the sensor’s output signal. The strain of the FBG does not affect the force compressing the PM-PCF either, so it should not affect the variable component of the sensor’s output signal. The FBG deformation determines the SLF operating point. If the operating point and the SLF transmission variations are within the linear portion of the SLF transmission around the Q point, the effect of the FBG strain on the variable component of the sensor output can be neglected. The proposed sensor operates in this range. To ensure such an operating regime, as mentioned above, the initial operating point of the SLF was shifted appropriately relative to the Q point. Outside of the linear operating range of the SLF, the FBG strain does affect the amplitude of the sensor output signal. The measurement ranges, which are determined by the peak spacing of the SLF intensity spectrum, can be increased or decreased by decreasing or increasing the length of the PM-PCF. However, there is a trade-off between measurement sensitivity and measurement range; the wider the measurement range, the lower the sensitivity and vice versa.

**Figure 8:**
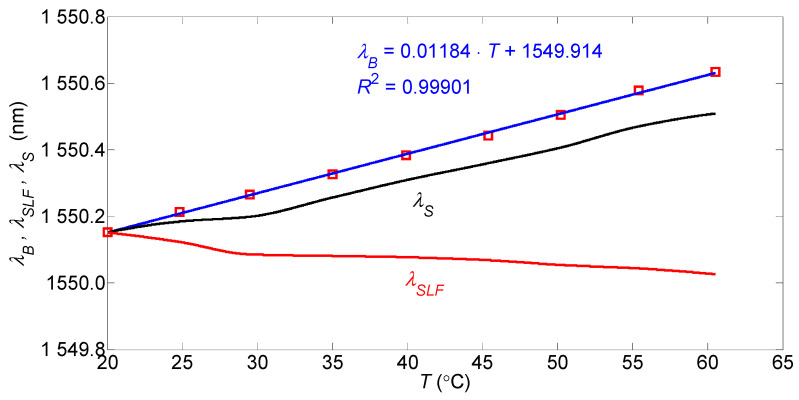
Center wavelength of the FBG reflection beam spectrum (*λ_B_*), the wavelength of the quadrature point of the SLF transmission spectrum (*λ_SLF_*), and the wavelength of the sensor output beam spectrum (*λ_S_*) as a function of temperature.

The sensor features a low temperature sensitivity. This is due to the opposite signs of the temperature sensitivities of the SLF relative to those ofthe PM-PCF and the FBG. The temperature sensitivity of the SLF with the PM-1550-01 fiber, in the range 20–30 °C, is about −6 pm/°C [26]. Measurements of the effect of temperature changes in the range 20–60 °C on the central wavelength of the employed FBG with recoating were carried out, the results of which are graphically presented in Figure 8.

They show that the FBG’s temperature sensitivity is 11.8 pm/°C. Therefore, the temperature sensitivity of the presented two-parameter sensor in the range (20–30) °C, in absolute terms, is almost the same as that of the SLF with the PM-1550-01 fiber. At higher temperatures, self-compensation is less effective due to the SLF’s lower absolute temperature sensitivity with the PM-PCF. The low-temperature sensitivity of the presented sensor is an important advantage.

Changes in ambient temperature affect the sensor’s constant component, shifting the SLF’s operating point. If changes in the operating point due to temperature are within the wavelength range where the SLF spectrum is linear, then temperature changes do not affect the accuracy of the variable force measurement. However, any change in the temperature causes an error inthe strain measurement. Taking into account the effect of partial temperature self-compensation and the strain sensitivity of the Bragg grating used, the value of the temperature cross-sensitivity of the strain measurement is equal to 4.8µε/°C. If this sensitivity is too high, one of the many methods used in temperature compensation of FBG sensors should be used. Treating the temperature compensation as a two-parameter measurement of strain and temperature, the analytical method presented in the introduction, whose detailed description is provided in references [1,2,3], can be used. An overview of internal and external FBG temperature compensation methods is available in many publications, such as [27,28].

## 4. Conclusions

A two-parameter fiber-optic sensor for simultaneous measurement of strain and time-varying force, and the results of measurements of its properties are presented. The sensor operates in a Sagnac loop filter configuration that uses simple intensity demodulation. For implementation, a narrowband reflected beam from a uniform FBG was used, illuminated by SLF. The FBG simultaneously acts as the strain-sensitive element. The sensor’s output voltage has a variable component and a constant component. The variable component is a measure of the time-varying transverse force acting on the PM-PCF, while the constant component is a measure of the tensile strain of the FBG. The linear operating range of the sensor is (0–4) N for force and (0–0.45) mε for strain, and its nonlinearity errors are 4.9% and 0.9% for force and strain, respectively. The proposed sensor features a simple design, no cross-sensitivities between the measurands in the linear operating range, low temperature sensitivity due to the partial temperature self-compensation as a result of opposite signs of the temperature coefficients of the SLF with the PM-PCF and the FBG, and the ability to demodulate a time-varying force and a quasi-static strain via DC/AC decomposition. The sensor can be used for testing composite materials and for technical diagnostics of structures made from them.

## Data Availability

Data are contained within this article.
